# Current situation, causes, and countermeasures to NIP vaccine shortages in Guangzhou, China

**DOI:** 10.1080/21645515.2019.1644883

**Published:** 2019-08-12

**Authors:** Zhiqun Li, Jindong Xu, Jianxiong Xu, Huifeng Tan, Chunhuan Zhang

**Affiliations:** a Guangzhou Center For Disease Control And Prevention, Guangzhou, China; b Department of Anesthesiology, Guangdong Cardiovascular Institute, Guangdong Provincial People’s Hospital & Guangdong Academy of Medical Sciences, Guangzhou, Guangdong, China

**Keywords:** NIP vaccine, shortage, cause, inventory reserve, countermeasures

## Abstract

From 2017 to 2018, Guangzhou experienced a shortage in 3–6 types of National Immunization Program (NIP) vaccines. To evaluate the current situation and causes of the NIP vaccine shortage, we analysed the duration, intensity and causes of the shortage from data in the vaccine information system of the Guangzhou Center for Disease Control and Prevention; we also proposed countermeasures to resolve the shortage. In 2017, there were shortages of three types of NIP vaccines in Guangzhou. The most affected vaccines were inactivated poliovirus (IPV) vaccine and meningococcal group AC (MenAC) vaccine, accounting for 39.5% and 16.5% of the reported shortages, respectively. In 2018, the shortage extended to six vaccine types; the most affected were diphtheria, tetanus and pertussis (DTP) vaccine, MenAC vaccine, and Bacille Calmette Guerin (BCG) vaccine. The four main causes for the shortage were: 1) an insufficient production capacity; 2) a delay in batch issuance; 3) vaccine-related events; and 4) an extended bidding procurement cycle. Four solutions are proposed: 1) expand production output; 2) exempt creditworthy enterprises from batch inspections; 3) establish alternative enterprises and emergency use authorizations; and 4) establish public health funds and stockpile storage systems.

## Introduction

Immunization is one of the most successful tools for the prevention of infectious diseases.^1^ Since 2007, the China National Immunization Program (NIP) vaccines have included the hepatitis B vaccine, Bacille Calmette Guerin (BCG) vaccine, polio vaccine, diphtheria and tetanus toxoids and acellular pertussis (DTaP) vaccine, measles vaccine, diphtheria-tetanus vaccine, hepatitis A vaccine, meningococcal polysaccharide vaccine, Japanese encephalitis vaccine, and measles-mumps-rubella (MMR) vaccine.^2^ Ensuring the supply of NIP vaccines is an important challenge for implementing the NIP. Vaccine shortages have been ubiquitous around the world. From May to October 2015, the Paediatric Tuberculosis Network European Trials Group (PTBNET) members surveyed 13 European countries; 8 countries reported BCG vaccine shortages (8/11.73%).^3^ The United States reported an MMR vaccine shortage in 2001, when 10.6 million doses were distributed (18.47% less than in 2000).^4^ Some vaccines were in short supply in China in 2016,^5^ resulting in public concerns about the insufficient supply of vaccines and a reduction in vaccination rates. Vaccine supply shortages have resulted in thousands of children who do not have complete vaccine-induced immunity throughout childhood in accordance with the NIP standards.

To date, China has not established a unified policy to address the challenges of vaccine shortages. The vaccine shortage was initially caused by an insufficient production output of vaccine manufacturing companies; these companies need to make adjustments by modifying or building new production lines with the assistance of supplementary vaccine production plans. EU countries usually select one candidate company if available, in addition to the primary bidder, to ensure adequate vaccine supply in case of insufficient output of the primary bidder.^6^ In 2008, the US military established an “emergency use authorization” mechanism^7^ in case of vaccine incidents or production stoppages or shortages. This mechanism allows authorization in the event of a vaccine shortage incident, and backup manufacturing companies will be authorized to invest in the production of vaccines that are in short supply. Batch issuance is one of the six national vaccine regulation functions required by the World Health Organization (WHO).^8^ Batch issuance has been generally proven to be an effective way to ensure the quality and safety of preventive public health vaccines. Since 2006, the vaccine batching and issuing system has been fully implemented.^9^ Each batch of vaccines must be tested; therefore, the workload at the testing and regulatory levels is substantial, and a production lag of a few months will affect the vaccine supply. The United States exempts products with stable performance and an excellent long-term track record from batch testing requirements.^10^

Currently, the Centers for Disease Control and Prevention (CDC) in each province in China accepts bids for NIP vaccine production within the province. Government funds are allocated into two batches every year, and the procurement is divided into two batches within a year. The procurement cycle is as long as 3–5 months. In July 2012, Australia used the National Health Funding Body (NHFB), a type of public health funding in the national health reform funding category, to purchase vaccines.^11^ There are specialized agencies and funds for vaccine procurement, which shortens the time allocated for procurement and streamlines the complicated procedures to facilitate vaccine supply. Building a vaccine stockpile is an effective policy response to surging vaccine demands and production disruptions, and the US CDC has funded the stockpile through Vaccines for Children (VFC) Program funding since 1983, with a target of 6 months inventory reserves.^12^ The use of NIP vaccine stocks is a good way to address vaccine shortages in China.

## Monthly number and duration of NIP vaccine shortages in 2017 and 2018

On May 1, 2016, China replaced the trivalent oral polio vaccine (TOPV) with the bivalent oral polio vaccine (BOPV) for use as the first and second doses for basic immunization. There is only one BOPV manufacturer (Beishengyan) in China. The supply shortage continued from 2016 to 2017, vaccine was unavailable in January and February. In March 2017, a normal supply was established, but in August and October, there was no vaccine distribution. Moreover, on May 1, 2016, the third round of the inactivated poliovirus (IPV) vaccination was also made available nationwide, but in February, June and September 2017, there was no IPV vaccine, and there was a six-month shortage. The meningococcal group A (MenA) vaccine shortage lasted for eight months from May to December 2017. The monthly shortage rate ranged from 17.9% to 38.4%. The meningococcal group AC (MenAC) vaccine shortage lasted seven months, with monthly shortages reaching 70.7%. The supply of the IPV and MenAC vaccines in 2018 increased from 2017, but the continuous shortage period also increased from 2017. The BCG vaccine was unavailable for 3 months, and the supply was short for 6 months. Measles and rubella (MR) and MMR vaccines were in short supply for 7 months. In July 2018, the Changchun Changsheng vaccine incidents occurred in China: the titers index of DTaP vaccine produced by Changchun Changsheng Biological Co., Ltd. was not up to standard (201605014-01 and 201605014-02 batches),The survey found that the batch number of 201605014-01 DTaP vaccine were distributed 252600 doses, all were distributed to Shandong Province; and the batch number of 201605014-02 DTaP vaccine were distributed 247200 doses, of which 223800 doses were distributed to Shandong Province and 23400 doses were distributed to Anhui Province, totaling 499,800 doses.^13^ The National Medical Products Administration ordered enterprises to stop production. Therefore, the supply of the DTaP vaccine in China is tight.The supply of DTaP vaccine began to decrease in August, and distribution was minimal. The shortage rates from August to December were 93.2%, 89.1%, 78.2%, 90.7%, and 99.5%, respectively. Only 500 doses were distributed in December (Table 1).

**Table 1.** NIP vaccine shortage rates by month in 2017 and 2018.

*NIP vaccine shortages were classified into two causes: the first was that the vaccine manufacturer was out of stock, and the CDC could not distribute the vaccine according to the plan in that month (defined as no vaccine); the second was that the vaccine manufacturer distributed only some or a few vaccines in proportion to the population in the whole province, and the CDC could not meet the needs of the plan in that month. We define the vaccines as vaccine shortages.

Red indicates no vaccine, yellow indicates a vaccine shortage and blue indicates normal supply. The number indicates the vaccine shortage rate. Monthly NIP vaccine shortage rate = (monthly planned NIP vaccine quantity – monthly distribute NIP vaccine quantity)/monthly plan NIP*100%.

Monthly planned NIP vaccine quantity: Annual Plan Number/12 (Month).

Monthly distribute NIP vaccine quantity: Number of vaccines distributed by provincial CDC every month.

## The extent of the NIP vaccine shortage and the causes for the shortages in 2017 and 2018

According to the vaccine management information system of the Guangzhou Centers for Disease Control and Prevention, in 2017 and 2018, all 249 vaccination clinics experienced shortages of two or more vaccines and 230 clinics experienced shortages of four or more NIP vaccines (Table 2). In 2018, there were 243 outpatient clinics that experienced vaccine shortages. In cases of vaccine shortages, the vaccines were allocated according to the number of children in the jurisdiction, and there was no stockpile. A total of 225 outpatient clinics reported that public complaints about the lack of vaccines increased by 30% from 2017; in 246 outpatient clinics, the IPV and MenAC vaccination rates decreased by 20–30%. In 2017 and 2018, 249 and 245 vaccination clinics, respectively, lacked vaccines for six months or longer.

**Table 2.** The extent of the NIP vaccine shortage in vaccination clinics in Guangzhou in 2017 and 2018.

Extent of NIP vaccine shortages	Number of vaccination clinics that reported NIP vaccine shortages	
	2017	2018
Shortages of two NIP vaccines	3	5
Shortages of three NIP vaccines	12	14
Shortages of four NIP vaccines	234	11
Shortages of five NIP vaccines		23
Shortages of six NIP vaccines		196
Subtotal	249	249
Shortages of six or more vaccines for 10 months or longer	249	2

There are many factors that can simultaneously affect the supply of vaccines. We have identified four key factors: 1) insufficient vaccine manufacturing capacity, 2) the titers index was not up to standard vaccine incident occurrence, 3) vaccine batch issuance restrictions, and 4) increased bidding procurement cycles.

The shortage of NIP vaccines in Guangzhou in 2017 and 2018 was unprecedented. Except for the BCG vaccine, all vaccine requirements were increased by the NIP in 2007; however, the production capacity for these vaccines was insufficient. In 2017, Wuhan Biology was the only manufacturer of the MenA vaccine. There were shortages due to insufficient production capacity and delays in wholesale distribution, and there were no backup suppliers. In 2017, there was a seven-month vaccine supply disruption in Guangzhou, and this long-term disruption increased the risk of an epidemic. Studies have shown that both the IPV and MenA vaccines were in short supply in 2017 and 2018, and frequent shortages of the same vaccine indicates a lack of effective regulation in vaccine manufacturing and procurement. In 2017, three vaccine shortages lasted 6–7 months; in 2018, six vaccine shortages lasted 5–10 months, which was an increase from 2017. In 2017, 234 vaccination clinics, accounting for 94.0% (234/249) of the city’s outpatient clinics, experienced a shortage of 4 varieties of NIP vaccines; in 2018, 78.7% (196/249) of outpatient clinics experienced shortages in 6 varieties of vaccines. The shortage was wide-ranging. In July 2018, the Changsheng vaccine incidents resulted in a shortage of DPT vaccine, with an annual shortage rate of 41.8% (753700/1296000) in Guangzhou. In July 1999, the United States proposed eliminating or reducing thiomersal in vaccines. The manufacturers of DTaP changed the packaging of vaccines from multi-dose vials to single-dose vials, reducing vaccine production by 25%. Moreover, a manufacturer decided to stop producing vaccines without prior notification, resulting in a shortage of DTaP vaccines.^14^ Establishing an emergency plan for vaccine shortage is a powerful measure for the government to alleviate the shortage of vaccines. A national public health fund is established to purchase vaccines. NIP vaccines are formed by centralized bidding by state organizations and the winning price is announced. Then, unified procurement is carried out by provinces, and procurement procedures are streamlined and cycle is shortened. Establish a stock reserve system to purchase NIP vaccine stocks for 6 months from qualified vaccine manufacturers (outside the annual plan). China has always attached great importance to the quality of vaccines. Since 2014, China has implemented the newly revised GMP requirements for vaccines and other aseptic production workshops.^15^ Vaccine production workshops that do not meet the requirements need to apply for certification and obtain the “GMP Certificate of Drugs” to produce vaccines. There are two BCG vaccine manufacturers in China, Chengdu and Shanghai Biology. From 2017 to 2018, some of their workshops need to be rebuilt and expanded to apply for GMP certification, resulting in a decline in vaccine production. In addition, due to various reasons, vaccine batch issuance restrictions, resulting in the validity of BCG vaccine listing for only three months. Vaccine production is complex and long, and the lack of new vaccines leads to a shortage of BCG vaccine. In 2017, Guangzhou distributed 56,000 doses, 394,600 doses in 2018 and 552,478 doses in 2018, with a vaccine shortage rate of 28.58% in 2018. The FDA promotes vaccine development and accelerated review through improved procedures; requires vaccine manufacturers to be informed six months in advance of their intention to stop producing vaccines; and increases the number of alternative vaccine products and suppliers as soon as possible to alleviate vaccine shortages.

The production of 14 types of NIP vaccines in China has been localized, with 7 state-owned enterprises and 6 private enterprises. The supply of NIP vaccines in China is generally fully self-sufficient. The occurrence of NIP vaccine shortages is due to vaccine production issues and tender procurement, which is not comprehensive enough. Additionally, a lack of timely emergency response to vaccine incidents and a lack of consideration of China’s implementation of the comprehensive two-child policy, which allows all couples can have two children as of January 2016, also contribute to vaccine shortages. The number of children who need to be vaccinated with free NIP vaccines has increased, and the vaccines are in short supply; there is no cooperation normalization between relevant departments.

## Conclusion

Our results show that the establishment of alternative enterprises is excluded in the procurement and bidding rules of the NIP in China. Designating only one primary producer may threaten the stability of the vaccine supply, and producing only 1–2 vaccine types can lead to insufficient production capacity and output. The procurement cycle is long, and the allocation of procurement funds is not timely and lacks flexibility. The relevant government departments are slow to respond to vaccine incidents; when vaccine-testing batches are delayed, the vaccine sampling is delayed, which affects the timing of release of the approved vaccine batches. All of these factors can contribute cumulatively to vaccine shortages.

This study shows that effective countermeasures to address the vaccine shortage are as follows: establish a special national public health fund to purchase vaccines, ensure the stable performance of vaccines produced by manufacturing enterprises, maintain good records of wholesale issuance, refine vaccine production plans, rebuild and build new production lines to improve production capacity, initiate emergency use authorizations in case of the titers index was not up to standard vaccine incidents, and create a stockpile.

## Abbreviations

Vaccine types
BOPV: bivalent oral poliovirus of serotypes 1 and 3
IPV: inactivated poliovirus
MenA: Meningococcal group A
MenAC: Meningococcal group AC
BCG: Bacille Calmette Guerin
DTaP: Diphtheria and tetanus toxoids and acellular pertussis
MMR: measles-mumps-rubella
